# The study of homology between tumor progression genes and members of retroviridae as a tool to predict target-directed therapy failure

**DOI:** 10.3389/fphar.2015.00092

**Published:** 2015-05-01

**Authors:** Janaina Fernandes

**Affiliations:** ^1^NUMPEX-BIO, Federal University of Rio de Janeiro, Duque de Caxias, Rio de Janeiro, Brazil; ^2^Institute for Translational Research on Health and Environment in the Amazon Region – INPeTAm, Federal University of Rio de Janeiro, Rio de Janeiro, Brazil

**Keywords:** target-directed therapy, oncoproteins, retrovirus, mutation rates, oncogenes, point mutation, drug resistance

## Abstract

Oncogenes are the primary candidates for target-directed therapy, given that they are involved directly in the progression and resistance of tumors. However, the appearance of point mutations can hinder the treatment of patients with these new molecules, raising costs and the need to development new analogs that target the novel mutations. Based on an analysis of homologies, the present study discusses the possibility of predicting the failure of a protein as a pharmacological target, due to its similarities with retrovirus sequences, which have extremely high mutation rates. This analysis was based on the molecular evidence available in the literature, and widely-used and well-established PSI-BLAST, with two iterations and maximum of 500 aligned sequences. The possibility of predicting which newly-discovered genes involved in tumor progression would likely result in the failure of targeted therapy, using free, simple and automated bioinformatics tools, could provide substantial savings in the time and financial resources needed for long-term drug development.

## The Impact of Point Mutations of Specific Targets on Drug Development

The resistance of cancers to chemotherapy may have a number of different determinants, which all lead to the same outcome—an increase in mortality rates. From the perspective of the development of new drugs, resistance may annul years of trials and the investment of millions of dollars. In some cases, the increase in patient survival may be well below expectations (Murphy and Stordal, 2011), and for many patients, the treatment may be totally ineffective. The cost of the development of a single drug, including clinical trials, has been estimated to reach as much as 800 million dollars, although it is likely to be even more than this, given that most companies do not disclose their operational data (DiMasi et al., 2003; Morgan et al., 2011).

Target-directed therapy offers a new approach that has a direct inhibitive effect on the molecules involved in the development of the cancer and its resistance to drugs (Neal and Sledge, 2014). The best documented case of targeted therapy in cancer molecular biology is that of the Tyrosine Kinase inhibitors (TKIs), which target the kinase domain of the BCR-ABL protein, which is responsible for the pathogenesis of chronic myelogenous leukemia (CML) through the enhancement of the proliferation and viability of myeloid cell lineage (Evans et al., 1993). Imatinib mersylate targets the kinase domain of BCR-ABL (Druker et al., 1996; Sawyers et al., 2002), and was designed to bind to the active site of the protein and block its biological activity. This approach revolutionized the treatment of CML, although new difficulties arose. The main drawback in target therapy is the mutation of the target, and in this specific case, the mutation of the imatinib binding site, which makes 20–40% of patients resistant to imatinib therapy (Shah et al., 2002; Azam et al., 2008). As the use of imatinib grew, case reports on imatinib resistant CML began to appear, indicating that the majority of resistant phenotypes are associated with point mutations in the BCR-ABL kinase domain (Roumiantsev et al., 2002).

The exact effects of mutations on the therapeutic efficiency of the TKIs are determined by the type of mutation (missense or silent) and their position in the target molecule (active site, P-loop). A number of studies have identified critical point mutations in the kinase domain of the BCR-ABL protein (Elias et al., 2014). These mutations reduce the affinity of the imatinib with its target, and may be accompanied by the amplification of the BCR-ABL gene (Gorre et al., 2001). Due to the clinical impact of the failure of imatinib therapy, the mutations were identified, and a second generation of the drug was developed to target these mutations (Weisberg et al., 2005).

This resulted in the development of Nilotinib, Dasatinib and Bosutinib, which improved the response in the patients, but once again, novel mutations arose, and recently, a third generation TKI, Ponatinib was approved (Santos et al., 2011). Treating imatinib-resistant patients with these new generation TKIs resulted in the appearance of *compound mutations*, i.e., multiple point mutations in the same allele, rather than multiple clones with different mutations (polyclonal mutations; Zabriskie et al., 2014). These tumor cells present a successive acquisition of mutations, and it was discovered that the identity of the component mutations present in a compound mutation reflects the type of drugs to which the patient was exposed previously (Khorashad et al., 2013).

The most frequent BCR-ABL mutation is T315I, which is known to cause resistance to most of the available TK inhibitors. The substitution of a threonine by an isoleucine impairs the formation of a hydrogen bond with the TKI, thus preventing the binding of the drug (Bose et al., 2013). Recent studies (Ferri et al., 2015) have shown that only the third generation TKI (ponatinib) is able to inhibit BCR-ABL with the T315I mutation. However, even ponatinib fails when facing compound mutations (Zabriskie et al., 2014).

A second group of drugs with a large set of *in vitro* and clinical data are the kinase inhibitors that target EGFR (Giaccone and Wang, 2011). A number studies have demonstrated the impact of point mutations in the targets on the outcome of the disease (Ahmed et al., 2014), as well as compound mutations (Berge et al., 2013), and enormous efforts have gone into the identification of mutations and the generation of analogs through *in silico* studies (Xiang et al., 2013; Banavath et al., 2014), giving rise to consecutive generations of the drugs.

An important feature common to all these targets is their high degree of homology with retroviral protein sequences. In fact, many of these human oncogenes have been named after their retroviral homologs. Examples include the human c-ABL, which is a homolog of v-ABL—the Abelson leukemia virus (Reddy et al., 1983), Src, a homolog of v- SRC—the Avian sarcoma virus (Schwartz et al., 1983), human AKT, a homolog of v-AKT—the AKT8 murine leukemia virus (Staal, 1987), and Kras, a homolog of the Kirsten murine sarcoma virus (Aaronson and Weaver, 1971).

Retroviruses are known to have high mutation rates, and one of the best documented is the mutation of the reverse transcriptase gene of the Human Immunodeficiency Virus, or HIV (García-Lerma and Heneine, 2001; Johnson et al., 2008). Due to a lack of correction mechanisms in the reverse transcriptase polymerization, the nucleotide substitution rate for retroviruses may reach 10^–4^ substitutions per cell infection (Preston et al., 1988), while in eukaryotes, due to their repair systems, these values are between 3 × 10^–8^ and 5 × 10^–11^ per cell division (Drake et al., 1998).

The present study hypothesizes that the similarity of human oncoproteins with retroviral sequences may have a major impact on the development of drugs for targeted therapy. In this case, the analysis of homologies between proposed targets and retroviral proteins may contribute to the identification of targets that are more likely to develop resistance to drugs designed for these targets. While the discussion of this hypothesis is based on the data available for BCR-ABL, it is hoped that the concept can be extended in practice to other oncogenes that present a high degree of homology with retroviruses.

## Materials and Methods

### Sequence Similarity

Sequence similarity was analyzed using PSI-BLAST—the Position Iterated-Local Alignment Search Tool (Altschul, 1997). The human form of each protein (*Homo sapiens* tax ID 9606) was aligned with those of viruses (tax ID:10239), with two iterations and a maximum of 500 aligned sequences. Artificial sequences and synthetic constructions were not considered for this analysis.

### Statistical Analysis

Statistical significance was generated via PSI-BLAST. A maximum E-value of 0.001 was considered for this analysis. The lower the E-value, the higher the homology.

## Hypotheses

### The Homology between Human Oncoproteins and Retroviral Proteins

When a new protein is identified as a drug target through molecular biology screenings, the structure may be elucidated by physical methods and/or *in silico* modeling. The latter is based on the identification of homologies between the target sequence and those known to occur in proteins which native structure were already elucidated (Floudas, 2007). Based on these results, the proteins are grouped in homolog families. These protein families are formed through gene duplication (Dharia et al., 2014 and Xu and Dumbrack, 2012).

In addition to vertical gene transfer through evolutionary processes, horizontal gene transfer may also occur. This process is responsible for the existence of endogenous retroviruses in the human genome (HERVs), which are believed to represent the footprint of ancient germ-cell retroviral infections. They would then have been transmitted vertically from one host generation to the next (de Parseval and Heidmann, 2005; Weiss, 2006), but most of them cannot produce an infectious virus due to the accumulation of mutations in key genes. Even so, some HERV genes have been adapted by the host genome for specialized functions, and some HERVs may be capable of inducing tumor progression (Serafino et al., 2009).

Exogenous retroviruses, in particular the acute transforming viruses, have also relied on horizontal gene transfer to acquire modifications of cellular genes and the capability of transforming animal cells. In comparison with the cellular version, viral oncogenes present mutations and deletions that alter the activity of the protein. Oncogenes involved in the development of human cancers were first identified in retroviruses, and for a time, it was believed that the origin of these oncogene was the oncoviruses. It was later revealed that the viral forms were cellular oncogenes acquired from the host by the retrovirus (Vogt, 2012). This discovery was based on the analysis of v-src, the viral form of the *Rous sarcoma virus*, the first oncovirus discovered (Vogt, 2012). In the specific case of BCR-ABL, its viral homolog, v-ABL, is present in the Abelson Murine Leukemia virus (A-MuLV) genome, which is thought to have originated from a recombination of the Muloney Murine Leukemia helper virus (M-MuLV) with a cellular sequence (*abl*) of the mouse genome (Reddy et al., 1983). As the viral version of an oncogene is a modified version of a mammalian sequence, a high degree of homology would be more than expected.

The search for homologies focused on the most prominent cases currently being researched for targeted therapy drug development, which, like BCR-ABL, belong to the tyrosine kinase family. Using PSI-BLAST with two iterations and a maximum of 500 sequences, the viral homologs were retrieved (Table 1). Most of them are truncated versions of the vertebrate protein, but they all conserve the domain necessary to transform cells. In this analysis, the lower the E-value, the greater the homology (Altschul, 1997).

**TABLE 1:** **Viral homologies found in human oncoproteins**.

Oncoprotein	Accession number of query sequence	E-value	Query coverage (%)	Taxon containing the homolog sequence	Viral group
Akt	NP_001014431.1	0	100	*AKT8 murine leukemia virus*	Retroviridae
Src	P12931.3	0	100	*Avian sarcoma virus S1*	Retroviridae
PI3K	NP_006209.2	0	98	*Avian sarcoma virus 16*	Retroviridae
ABL	NP 005148.2	0	72	*Abelson murine leukemia virus*	Retroviridae
RAF	P04049.1	0	57	*IC4 retrovirus*	Retroviridae
EGFR	NP 005219.2	0	52	*Avian leukosis virus*	Retroviridae
ROS1	NP 002935.2	1^–162^	62	*UR2 sarcoma virus*	Retroviridae
FGFR1	NP_075598.2	6^–151^	81	*Feline sarcoma virus*	Retroviridae
RET	P07949.3	5^–146^	39	*UR2 sarcoma virus*	Retroviridae
MET	NP 001120972.1	1^–131^	23	*Avian erythroblastosis virus*	Retroviridae
KRAS	NP 004976.2	3^–122^	100	*Kirsten murine sarcoma virus*	Retroviridae
IGFR1	NP 000557.1	5^–122^	33	*Avian sarcoma virus S1*	Retroviridae
DDR	NP 001945.3	2^–115^	38	*Avian sarcoma virus S1*	Retroviridae
FAK	Q05397.2	5^–51^	28	*Feline sarcoma virus*	Retroviridae
IRAK1	AAH54000.1	2^–86^	46	*Acanthamoeba poliphaga mimivirus*	Mimiviridae (dsDNA vírus)

In these and other oncogenes, mammal sequences are incorporated into the viral genome, and thus become subject to the mutation rates of retroviruses. During multiple cycles of virus infection, mutants are produced as a result of the poor fidelity of retroviral reverse transcriptases. Mutations are also induced in the captured sequence, altering it, and in some cases activating its ability to transform the target cells. A question that arises here is whether the similarities in the sequences also extend to their mutational status?

### Mutation Rates in Retroviruses and Tumors

Mutation rates (μ) can be measured as substitutions per nucleotide per cell infection (in viruses) and substitutions per nucleotide per replication cycle in eukaryotes. The size of the genome is also an important parameter here (Drake and Holland, 1999). A number of studies have shown that the retroviruses have one of the highest mutation rates of all viruses (Sanjuán et al., 2010). Sequences subject to high mutation rates provide a broad spectrum of random mutants for adaptation to environmental changes, which is advantageous in general (Loeb et al., 1974). The capacity of tumors and viruses generate and select viable mutants will be proportional to their mutation and replication rates (Fox and Loeb, 2010). While normal cells have mutation rates of around 10^–11^ (Table 2), this rises to approximately 10^–7^ in tumor cells, which is higher than that found in unicellular eukaryotes and dsDNA viruses. Retroviruses have mutation rates around 10^–5^, in other words, just two orders of magnitude greater than that found in tumors, whereas the difference is of four orders of magnitude between normal and tumor cells, and six orders of magnitude between normal cells and retroviruses.

**TABLE 2:** **Mutation rates expressed as substitutions per nucleotide per cell infection (in viruses) and mutations per cell division (in eukaryotes–yeast, tumor and normal cells)**.

Group	μ	Reference
dsRNA (Retroviruses)		
Murine leukemia virus	3.0 × 10^–5^	Sanjuán et al. (2010)
Human T-Cell leukemia virus	1.6 × 10^–5^	Sanjuán et al. (2010)
type 1 (HTLV1)		
Bovine leukemia virus	1.7 × 10^–5^	Sanjuán et al. (2010)
Human immunodeficiency virus	2.4 × 10^–5^	Sanjuán et al. (2010)
type 1 (HIV1)		
Rous sarcoma virus (RSV)	1.4 × 10^–4^	Sanjuán et al. (2010)
Spleen necrosis virus (SNV)	3.7 × 10^–5^	Sanjuán et al. (2010)
dsDNA		
Herpes simplex virus type 1	5.9 × 10^–8^	Sanjuán et al. (2010)
Bacteriophage T2	9.8 × 10^–8^	Sanjuán et al. (2010)
Yeast		
MSH2 WT	4.8 × 10^–10^	Lang et al. (2013)
MSH2 null (repair deficient)	7.1 × 10^–8^	Lang et al. (2013)
Tumor cells		
MM96L (melanoma)	2.1 × 10^–7^	Musk et al. (1996)
Normal cells	5 × 10^–11^	Drake et al. (1998)

Despite this variation in mutation rates, functional versions of the same oncoprotein (for example, Src) can be found in normal (unmutated; Knoll and Drescher, 2004; Francis et al., 2015), in tumors (mutated; Hong et al., 2014) and in retrovirus (truncated and mutated; Masker et al., 2007; Vogt, 2012).

These mutation rates are reflected in the rapid emergence of resistance to therapy in tumor cells and viruses, and it has even been suggested that this characteristic could be exploited therapeutically through *lethal mutagenesis* (*Mutational catastrophe*). In this approach, nucleotide analogs are used to enhance the mutation rate of a virus (Baranovich et al., 2013) and tumor cells (Fox and Loeb, 2010) beyond the threshold of error catastrophe, leading them to extinction (Fox and Loeb, 2010). The deleterious effects of high mutation rates may nevertheless be counteracted by mutational robustness, i.e., the constancy of a phenotype in the context of deleterious mutations (Sanjuán et al., 2007). In fact, the mutational robustness of RNA viruses is higher than that of other viruses and may thus have a negative effect on their sensitivity to lethal mutagenesis (Graci et al., 2012).

**FIGURE 1:**
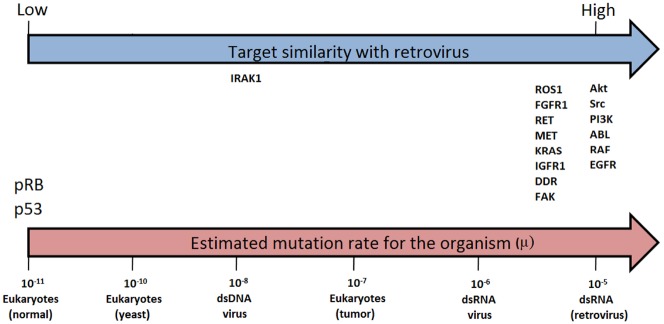
**Similarity between retroviral and evaluated target sequences (blue arrow) and estimated mutation rates (μ) for selected organisms (μ = substitutions per nucleotide per cell infection in viruses and per cell division in eukaryotes, i.e., yeast, tumor and normal cells).** Estimates from Sanjuán et al. (2010; pink arrow).

The constancy of the phenotype (the induction of cell transformation through kinase activity) under high mutation rates, is actually being tested continuously, given that highly homologous sequences of the oncogenes are present in the highly proliferative retroviruses which can successfully infect and transform normal cells (Masker et al., 2007). In this case, if a viral sequence has a mutation rate around six orders of magnitude higher than the same sequence in a normal human cell, during the proliferation of the tumor, the retroviral homologs will be mutating at a much higher rate than a normal cell, but below the catastrophe threshold. Thus, the high mutation rate, coupled with high proliferation and the selective environment created by target directed drugs will result in increasing adaptation, rather than extinction. From the viewpoint of the drug discovery, then, a target that is highly homologous with a retrovirus is likely to be resistant to drugs, and will probably result in the failure of the drugs developed for new targets during the first generation (Figure 1).

### Targets Presenting Variable Degrees of Homology with Retroviruses

As discussed by Fernandes et al. (2012), sequences in tumors that are highly homologous to those of retroviruses are typical of drug resistance, and will contain point mutations that confer resistance on the tumors containing them. On the other hand, sequences of tumor-suppressing genes, such as p53 and pRB, that are not homologous to any retrovirus family, will accumulate mutations, and may cease to function under high mutation rates. This implies that the degree of homology between a sequence and a viral group may reflect its robustness.

Recent studies have identified a number of molecules as candidates for the development of drugs (Alamgeer et al., 2013). These include RET (Qi et al., 2011), ROS1 (Sun et al., 2014), c-MET (Kim et al., 2014), and DDR1 (Kim et al., 2013), all of which are currently being trialed (Frett et al., 2014). All these molecules present a degree of similarity with a retroviral protein (Table 1). For example, RET is homologous with the P68 protein of the UR2 sarcoma virus, while ROS1 is a homolog of v-Ros from the avian UR2 sarcoma virus oncogene (Matsushime et al., 1986). Similarly, c-MET is a homolog of v-Sea from the Avian Erythroblastosis Virus (Smith et al., 1989), and DDR1 a homolog of v-Src from the Avian Sarcoma Virus. In some of these cases, resistance to targeted therapy has already been recorded (Sun et al., 2014). However, the higher homology of one other emerging target, IRAK1 (interleukin-1 receptor-associated kinase 1) was found not with a retroviral sequence (Table 1), but one belonging to a dsDNA virus (*Acanthamoeba polyphaga mimivirus*).

IRAK1 is involved in IL-1beta-induced NF-kappaB activation (Chen et al., 2002), and also in the reduced apoptosis in myelodysplastic syndrome (Rhyasen et al., 2013). The IRAK1 mutation has been found in Primary effusion lymphoma (PEL), a disease that develops in HIV-positive patients, in association with viral infections (Yang et al., 2014). Only additional data will confirm whether IRAK1 presents point mutations under pressure from its inhibitors.

### Could it be Possible to Predict Which Mutations Would Appear in a Cancer Target Prior to Clinical Evaluation?

The mutational status of a sequence is determined by its mutation and proliferation rates. But exactly which amino acid may be substituted in a given position (such as T315I in the case of BCR-ABL), and its importance for the outcome of a chemotherapy procedure, is related to selection of which particular mutation will confer advantage (Leder et al., 2011). Human oncoproteins and retroviral protein sequences could theoretically present similar pattern of mutations if both sequences present the proliferation rates and are affected by the same selection pressures. In the case of imatinib and its analogs targeting BCR-ABL, the same mutations are selected for, and the results may even reflect the order in which the drugs were applied (Shah et al., 2007; Khorashad et al., 2013). Obviously, highly similar sequences subject to similar replication rates, replicated by the same genetic system and similar selection pressures will likely present the same pattern of mutation.

As discussed above, samples obtained from patients can be used to identify the most frequent and relevant mutations, but the question remains as to whether it may be possible to identify relevant mutations in a specific target during the development of a new drug. Using an *in vitro* approach, Joseph et al. (2013) were able to identify mutations that conferred resistance to second-generation antiandrogens. The same mutations were also found in samples from the patients being treated, corroborating the clinical relevance of this particular mutation.

The *in vitro* prediction of the identity of a mutation that likely confers resistance to a new drug without concomitant corroboration by clinical trials would be an economically attractive prospect, although the direct application of these results for drug development it would be an issue. However, the possibility of providing an inventory of potentially relevant point mutations, which could be investigated further through *in silico* studies, might represent a potentially economic strategy.

## Conclusion

The interchange with evolutionary models has contributed to the understanding of cancer as a highly complex disease. In cancer research, the ongoing integration of molecular biology with disciplines such as theoretical ecology (Korolev et al., 2014), mathematics (Beerenwinkel et al., 2015), and evolutionary biology (Greaves, 2009) has provided important practical applications for the discovery of new drugs.

Some proteins that possess less similarity with sequences resident in retroviruses and are relevant for tumor development, are those that act suppressing cancer, as p53 and pRB. These molecules appeared late in evolution to allow the establishment of the multicellular organisms (Koonin and Aravind, 2002). Those sequences are less robust since high mutation rates abrogate their function. As shown in Figure 1 these sequences rely on the opposite side of oncogenes regarding similarity with retrovirus.

Most of the targets for drug development are molecules involved in tumor progression, exactly because these sequences maintain their function under high mutation rates. The hypotheses presented here give rise to a challenge: identify a gene that promotes enough tumor progression to be considered a pharmacological target. At the same time, this sequence must be subjected to low mutations rate. The later feature may be reflected by its similarity with retroviral sequences.

In the context of the present study, another consideration arises—if it is possible to identify a point mutation that potentially reduces the efficiency of a new drug prior to clinical trials, should this influence the decision to develop the drug further? In other words, should the development of the drug continue if it is known that it will fail in some degree due to the similarity of the target sequence with retroviral proteins?

There are good reasons to proceed, however. In the specific case of the application of TK inhibitors to the treatment of leukemia and lung cancer treatment as examples, diseases that target sequences have viral homologous, one important reason is that most patients (70–80%) respond to targeted therapy. This represents some 246,000 leukemia patients and 1,277,500 lung cancer patients, worldwide, each year (Ferlay et al., 2015). In addition, the prior *in vitro* identification of the mutants that will be resistant to the first generation of a drug would contribute to the improvement of therapeutic strategies for non-responding patients. This would hopefully contribution to acceleration in the inevitable development of subsequent generations of the new drug.

### Conflict of Interest Statement

The author declares that the research was conducted in the absence of any commercial or financial relationships that could be construed as a potential conflict of interest.
